# Aire-Overexpressing Dendritic Cells Induce Peripheral CD4^+^ T Cell Tolerance

**DOI:** 10.3390/ijms17010038

**Published:** 2015-12-29

**Authors:** Dongbei Li, Haijun Li, Haiying Fu, Kunwei Niu, Yantong Guo, Chuan Guo, Jitong Sun, Yi Li, Wei Yang

**Affiliations:** Department of Immunology, Norman Bethune College of Medicine, Jilin University, Changchun 130021, China; lidongbei2008@163.com (D.L.); 13944883280@163.com (H.L.); fuhy@jlu.edu.cn (H.F.); niukw123@163.com (K.N.); gb123450000@163.com (Y.G.); guochuanxy@163.com (C.G.); sjitong@163.com(J.S.)

**Keywords:** Aire, DCs, CD4^+^T cells, T1D

## Abstract

Autoimmune regulator (Aire) can promote the ectopic expression of peripheral tissue-restricted antigens (TRAs) in thymic medullary epithelial cells (mTECs), which leads to the deletion of autoreactive T cells and consequently prevents autoimmune diseases. However, the functions of Aire in the periphery, such as in dendritic cells (DCs), remain unclear. This study’s aim was to investigate the effect of Aire-overexpressing DCs (Aire cells) on the functions of CD4^+^ T cells and the treatment of type 1 diabetes (T1D). We demonstrated that Aire cells upregulated the mRNA levels of the tolerance-related molecules *CD73*, *Lag3*, and *FR4* and the apoptosis of CD4^+^ T cells in STZ-T1D mouse-derived splenocytes. Furthermore, following insulin stimulation, Aire cells decreased the number of CD4^+^ IFN-γ^+^ T cells in both STZ-T1D and WT mouse-derived splenocytes and reduced the expression levels of TCR signaling molecules (Ca^2+^ and p-ERK) in CD4^+^ T cells. We observed that Aire cells-induced CD4^+^ T cells could delay the development of T1D. In summary, Aire-expressing DCs inhibited TCR signaling pathways and decreased the quantity of CD4^+^IFN-γ^+^ autoreactive T cells. These data suggest a mechanism for Aire in the maintenance of peripheral immune tolerance and provide a potential method to control autoimmunity by targeting *Aire*.

## 1. Introduction

In the thymus, T cells develop, mature and form a polyclonal T cell repertoire capable of identifying large numbers of exogenous pathogens to prevent the occurrence of diseases. During this process, T cell clones that recognize self-antigens are inevitably generated. To maintain self-tolerance of a normal body, T-cell clones that react with self-antigens need to be depleted [1]. Autoimmune regulator (Aire)-mediated thymic negative selection is the main mechanism underlying the elimination of autoreactive T cells by the central immune system [2]. However, a portion of the autoreactive T cells may escape negative selection and enter into the periphery. To prevent the occurrence of autoimmune diseases, further elimination or tolerance induction of the autoreactive T cells in the periphery is necessary [3,4,5]. Studies have shown that peripheral splenic and lymph node stromal cells and dendritic cells (DCs) play important roles in the clearance of autoreactive T cells that have escaped thymic negative selection and entered the periphery [6,7,8].

Among the central immune organs, *Aire* is mainly expressed in medullary thymic epithelial cells (mTECs) [9]. Aire regulates the expression of a variety of tissue-restricted antigens (TRAs) and mediates the clearance of autoreactive T cells. Additionally, Aire induces the production of regulatory T cells (Tregs), thereby maintaining central immune tolerance [2,10,11,12]. Loss or mutation of the *Aire* gene causes autoimmune polyglandular syndrome type I (APSI) [13], which is also known as autoimmune polyendocrinopathy-candidiasis-ectodermal dystrophy (APECED). The clinical characteristics of APSI are presented as autoimmune diseases involving multiple glands, such as Addison’s disease, hypothyroidism, thyroid disease and type 1 diabetes (T1D) [14,15].

Recently, *Aire* expression has also been observed in peripheral tissues, especially in peripheral blood and lymph node-derived DCs, macrophages, and epithelial cells. However, the role of Aire in peripheral tissues is poorly understood [9,16,17,18]. Our previous study has shown that *in vitro*, macrophages overexpressing Aire elevated a subset of CD4^+^Foxp3^+^ Treg cells [19]; however, the effect on the treatment of autoimmune diseases needs further research. Studies have shown that Aire regulates the expression of certain TRAs on antigen-presenting cells (APCs), such as myelin oligodendrocyte glycoprotein (MOG) and insulin, thereby playing a role in the clearance of peripheral autoreactive T cells [20]. Bone marrow-derived mononuclear cells transduced with *Aire* are capable of delaying the occurrence of MOG-induced experimental autoimmune encephalomyelitis (EAE) [21]. Moreover, insulin autoantigen is mainly expressed on Aire^+^ DCs in the spleen [22]. A study of non-obese diabetic (NOD) mice showed that decreased expression of pancreatic tissue-associated antigens in the peripheral lymph nodes aggravated the severity of the disease [23,24,25,26,27,28]. Therefore, we speculated that Aire prevented the development of autoimmune diseases such as T1D by inducing peripheral autoreactive T cell tolerance through the regulation of the expression of related molecules and TRAs on DCs.

In the present study, we utilized the Aire-overexpressing DC cell line DC2.4 to examine the effect of Aire on molecules related to DC tolerance. Based on these findings, the Aire-overexpressing DC cell line DC2.4 was co-cultured with splenocytes derived from mice with streptozotocin (STZ)-induced T1D to examine the effect of Aire-overexpressing DCs on the tolerant status of CD4^+^ T cells. Furthermore, the mechanism by which Aire-overexpressing DCs induced the functional inactivation of CD4^+^ T cells was explored. Finally, the effects of CD4^+^ T cells induced by Aire-overexpressing DCs on the incidence of T1D in mice were examined. The results showed that Aire induced tolerance in T1D-related autoreactive T cells and prevented the occurrence of T1D by regulating the expression of cell surface molecules and T1D-associated TRAs in DCs.

## 2. Results and Discussion

### 2.1. The Effect of Aire on Molecules Related to DC Tolerance

**Figure 1:**
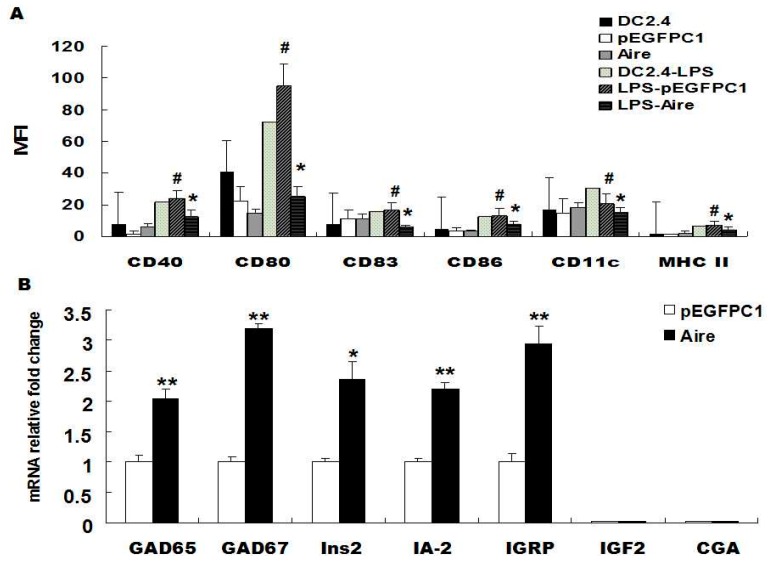
Aire affected the maturation of DC2.4 and its TRA expression levels. (**A**) The expression of CD40, CD80, CD83, CD86, CD11c and MHC II were detected by FACS in Aire and control cells with or without stimulation with 10 μg/mL of LPS for 48 h; (**B**) GAD65/67, Ins2, IA-2, IGRP, IGF2, and CGA levels were detected by RT-qPCR in Aire and control cells. The data are shown as the expression levels relative to the expression of GAPDH and are depicted as the fold changes relative to the control cells normalized to 1. The data are expressed as the means±SD from three to five independent experiments. ^#^ *p* < 0.05, LPS stimulation *vs.* control; * *p* < 0.05, Aire *vs.* pEGFPC1, ** *p* < 0.01,Aire *vs*. pEGFPC1.

Studies have shown that immature DCs maintain tolerance through the expression of low levels of related cell surface molecules [29]. Therefore, to investigate whether Aire could maintain the immature state of DCs, we examined the expression of cell surface molecules on unstimulated and lipopolysaccharide (LPS)-stimulated Aire cells. The results showed that the expression of CD40, CD80, CD83, CD86, CD11c and MHC-II was significantly lower in Aire cells stimulated with 10 μg/mL of LPS for 48 h compared to their expression levels in the control cells. No differences were observed in the expression levels of CD40, CD80, CD83, CD86, CD11c and major histocompatibility complex class *II* (MHC II) between the two groups of unstimulated cells (Figure 1A). The results were similar at 24 h post stimulation with LPS (Supplementary Material, Figure S1). TRAs expressed in peripheral lymph nodes have been reported to be related to the clearance of autoreactive T cells and the maintenance of immune tolerance [7,8]. To verify the effects of Aire on the expression of T1D-related TRAs in DCs, we examined the mRNA expression levels of T1D-associated TRAs on Aire-expressing cells by quantitative reverse transcription polymerase chain reaction (RT-qPCR). The results showed that the mRNA levels of *glutamic acid decarboxylase 65/67* (*GAD65/67*), *insulin*-*2* (*Ins2*), *insulinoma antigen 2* *(IA-2)* and *islet-specific glucose-6-phosphatase catalytic subunit-related protein (IGRP)* were significantly elevated in the Aire cells compared with the control cells. The expression of *insulin-like growth factor 2 (IGF2)* and *chorionic gonadotropin alpha chain (CGA)* was not detected in either the Aire or control cells (Figure 1B). In summary, Aire is one of the factors that maintains the immature state of DCs with or without stimulation by LPS and promotes the expression of T1D-related TRAs in DCs.

### 2.2. The Effect of Aire-Overexpressing DCs on the Tolerant Status of CD4^+^ T Cells

**Figure 2:**
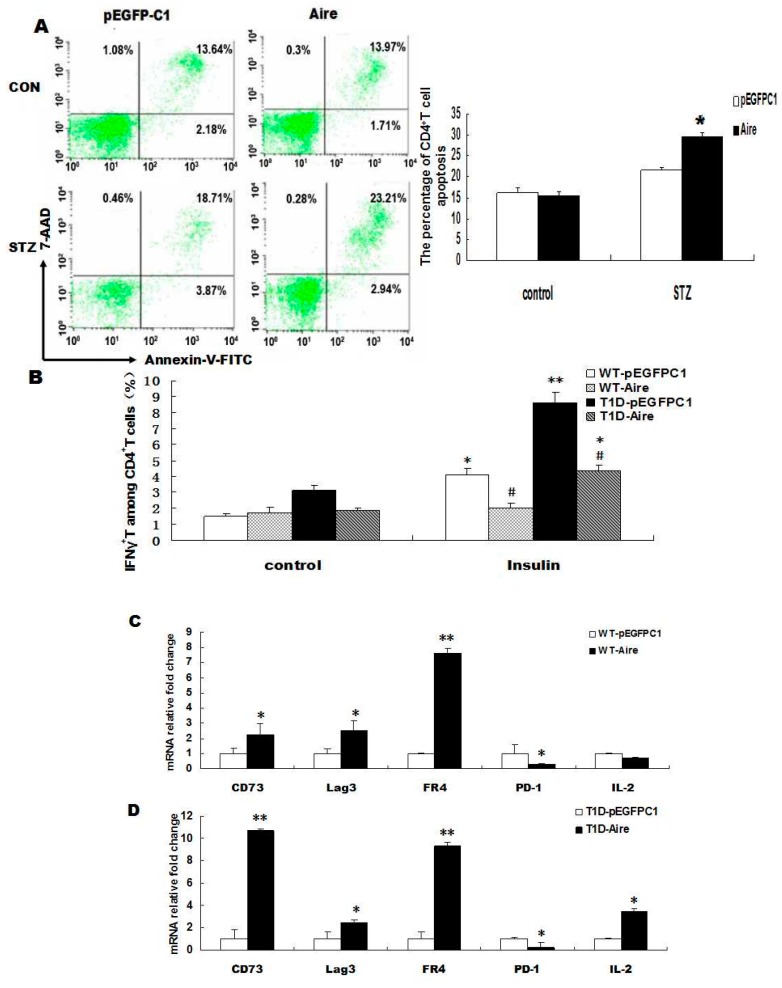
Aire cells altered the tolerance status of CD4^+^ T cells. (**A**) Splenocytes from STZ-induced T1D mice and WT mice co-cultured with Aire or control cells for 48 h. The apoptosis of CD4^+^ T cells was analyzed by FACS using the Annexin V-7AAD kit; (**B**) Splenocytes from STZ-induced T1D mice or WT mice co-cultured with Aire or control cells for 48 h in the presence of insulin. The IFN-γ expression levels in CD4^+^ T cells were examined by FACS; (**C**,**D**) Splenocytes from STZ-induced T1D mice and WT mice co-cultured with Aire or control cells for 48 h. The CD73, Lag3, FR4, PD-1 and IL-2 levels were detected by RT-qPCR in the splenocytes. The results are presented as the percentage of positive cells ± SD (*n* = 5); each experiment was repeated at least three times. ^#^ *p* < 0.05, Insulin stimulation *vs.* control; * *p* < 0.05, ** *p* < 0.01, Aire *vs.* pEGFPC1.

DC cells are capable of inducing the apoptosis of autoreactive CD4^+^ T cells or functionally inactivating autoreactive CD4^+^ T cells [3,4,5], thereby reducing the production of interferon γ (IFN-γ), maintaining self-tolerance and eventually preventing the occurrence of autoimmune diseases. To investigate whether Aire-overexpressing DC2.4 cells impacted the apoptosis of autoreactive CD4^+^ T cells, splenocytes derived from mice with STZ-induced T1D (STZ-T1D) and wild type (WT) mice were co-cultured with either Aire or control cells for 48 h. Then, we examined CD4^+^ T cells apoptosis by flow cytometric analysis. The results showed that the apoptotic rate of STZ-T1D mouse-derived CD4^+^ T cells was higher in the Aire group compared to the control group. In contrast, no significant difference was detected in the apoptotic rates of WT mouse-derived CD4^+^ T cells between the two groups (Figure 2A). These results demonstrated that Aire-overexpressing DCs induced the apoptosis of autoreactive CD4^+^ T cells, suggesting that Aire DCs might induce CD4^+^ T cell tolerance.

Next, we explored whether Aire-overexpressing DC2.4 cells induced functional inactivation of CD4^+^ T cells. Splenocytes derived from STZ-T1D and WT mice were co-cultured with either Aire cells or control cells for 48 h and then stimulated with insulin for 24 h. The STZ-T1D mouse and WT mouse-derived splenocytes from the Aire group had significantly reduced numbers of CD4^+^ IFN-γ^+^ T cells compared to the splenocytes from the control group (Figure 2B, Figure S2). These results preliminarily indicated that Aire-transfected DCs might induce functional inactivation of CD4^+^ T cells. However, the activation markers and other type of cytokines involved need to analyzed in future studies.

To confirm the tolerant status of CD4^+^ T cells after induction by Aire-transfected DCs, we examined the mRNA expression levels of tolerance-related surface molecules by RT-qPCR, including CD73, lymphocyte-activation gene 3 (Lag3), folate receptor 4 (FR4) and programmed cell death protein 1 (PD-1), and the mRNA expression level of the cytokine interleukin 2 (IL-2) in T cells after co-culturing splenocytes and Aire cells [30,31]. The results showed that the mRNA expression levels of CD73, Lag3, FR4 and IL-2 were significantly higher in STZ-T1D mouse-derived splenocytes from the Aire group compared with those from the control group. In contrast, the expression of PD-1 was markedly decreased in the Aire group compared with the control group (Figure 2D). The mRNA expression levels of CD73, Lag3 and FR4 were also higher in WT mouse-derived splenocytes from the Aire group, whereas PD-1 expression was lower in the Aire group compared to the control group. One possible explanation for the low expression of PD-1 in splenocytes from the Aire group may be related to PD-1’s dual role in T cells (Figure 2C). In summary, Aire-transfected DCs promoted the apoptosis of STZ-T1D mouse-derived CD4^+^ T cells, reduced the number of IFN-γ-producing autoreactive CD4^+^ T cells in STZ-T1D and WT mouse and upregulated the expression of tolerance-related molecules in splenocytes, suggesting that Aire-overexpressing DCs may play a role in maintaining the tolerant state of CD4^+^ T cells.

### 2.3. The Mechanism by Which Aire-Overexpressing DCs Induces Functional Inactivation of CD4^+^ T Cells

**Figure 3:**
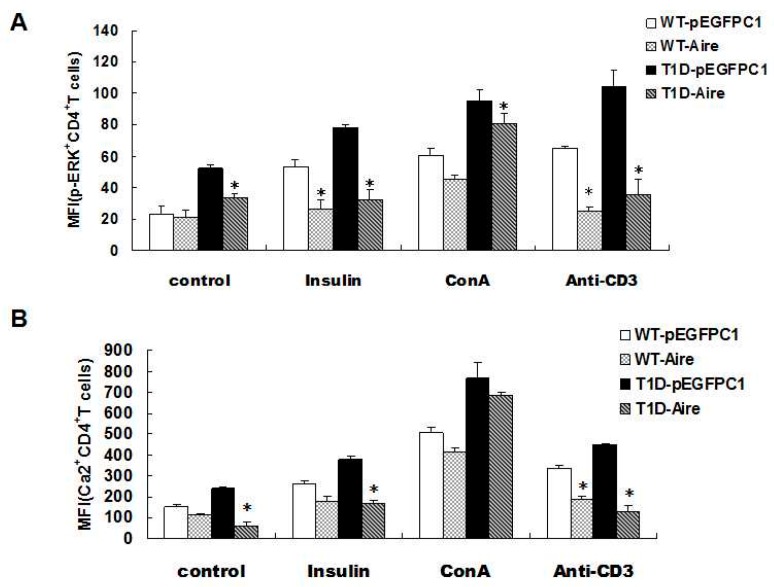
Aire cells affected TCR signaling in CD4^+^ T cells among the splenocytes from STZ-T1D mice. The splenocytes from STZ-induced T1D mice and WT mice co-cultured with Aire or control cells in the presence of insulin, ConA or an anti-CD3 antibody for 48 h. (**A**) The p-ERK expression levels; and (**B**) the concentration of Ca^2+^ in CD4^+^T cells were examined by FACS. The results are presented as the average percentage ± SD (*n* = 5); each experiment was repeated at least three times. * *p* < 0.05, Aire *vs.* pEGFPC1.

The T cell receptors (TCRs) located on the surface of the autoreactive T cells recognize the major histocompatibility complex class II (MHC II) complexes formed after DC-mediated antigen presentation. Antigen recognition activates the intracellular TCR signaling pathways and promotes the production of IFN-γ by activated T cells, resulting in autoimmune diseases [32]. Therefore, the next issue to explore was whether Aire cells influenced IFN-γ production in CD4^+^ T cells derived from STZ-T1D mice by affecting TCR signaling pathways. STZ-T1D and WT mouse-derived splenocytes were co-cultured with either Aire cells or control cells and then stimulated with an anti-CD3 antibody, concanavalin A (ConA) or insulin. Subsequently, we examined the key molecules of the TCR signaling pathways in CD4^+^ T cells *by* fluorescence-activated cell sorting (FACS), including Ca^2+^ and phosphorylated extracellular signal-regulated kinase (p-ERK) [33]. The results showed that the Ca^2+^ and p-ERK levels after stimulation with the CD3 antibody and insulin were lower in the STZ-T1D and WT mouse-derived CD4^+^ T cells from the Aire group compared with those from the control group. Although the levels of Ca^2+^ and p-ERK were elevated in STZ-T1D and WT mouse-derived CD4^+^ T cells upon stimulation with the unrelated control ConA compared to the unstimulated CD4^+^T cells, there were no significant difference in the levels of Ca^2+^ and p-ERK between the Aire and control groups after ConA stimulation (Figure 3A,B, Supplementary Material, Figure S3). These results indicated that Aire-overexpressing DCs reduced the mRNA expression levels of key molecules of the TCR signaling pathways (Ca^2+^ and p-ERK) in STZ-T1D mouse-derived CD4^+^ T cells, resulting in the functional inactivation of autoreactive CD4^+^ T cells. Thus, this may be one pathway by which Aire induces tolerance in autoreactive T cells.

### 2.4. The Effects of CD4^+^T Cells Induced by Aire-Overexpressing DCs on the Incidence of T1D in Mice

**Figure 4:**
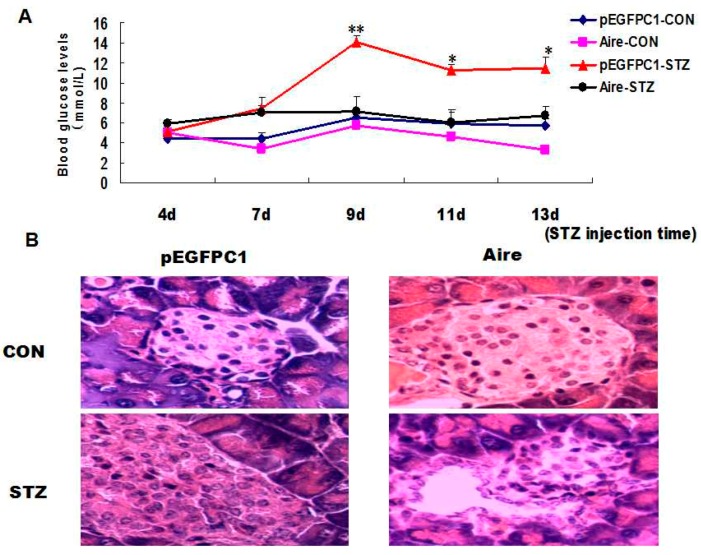
Adoptive transfer of Aire cell-co-cultured splenocytes delayed the development of type 1 diabetes. Splenocytes from WT mice were co-cultured with Aire or control cells for 48 h. Then, the lymphocytes were collected and injected into WT mice by the tail vein, followed by the induction of diabetes with STZ. (**A**) The blood glucose levels in mice transferred spleen lymphocytes; (**B**) the histology of pancreas of mice transferred spleen lymphocytes with H&E staining, *n* = 5 in each group, * *p* < 0.05, ** *p* < 0.01, Aire *vs*. pEGFPC1; Original magnification, ×200.

Our previous study demonstrated that the expression levels of IFN-γ, Ca^2+^ and p-ERK were markedly reduced in CD4^+^ T cells after splenocytes derived from WT mice were co-cultured with Aire cells and stimulated with insulin. These results led us to speculate whether Aire-induced CD4^+^ T cells would inhibit or delay the occurrence of T1D in STZ-induced mice. In the following experiment, splenocytes derived from WT mice were co-cultured with Aire cells or control cells for 48h. The purity (Supplementary Material, Figure S4A) and the rate of apoptotic T-cells (Supplementary Material, Figure S4B) were detected. A low percentage of aptoptotic T cell was detected in each group, and there was no difference between the Aire-expressing DC group and control group. Then the splenocytes were intravenously injected into WT mice through the *tail vein*. Subsequently, we established a mouse model of T1D induced with STZ and monitored the blood glucose levels. The results showed that the T1D model mice developed typical symptoms. However, the blood glucose levels were significantly lower in mice transplanted with splenocytes of the Aire group compared to those in mice transplanted with splenocytes of the control group (Figure 4A). After two weeks, the pancreatic tissues were collected and subjected to hematoxylin and eosin (H & E) staining. The degree of inflammatory infiltration was significantly lower in the pancreatic tissues of mice transplanted with splenocytes from the Aire group (Figure 4B).

**Figure 5:**
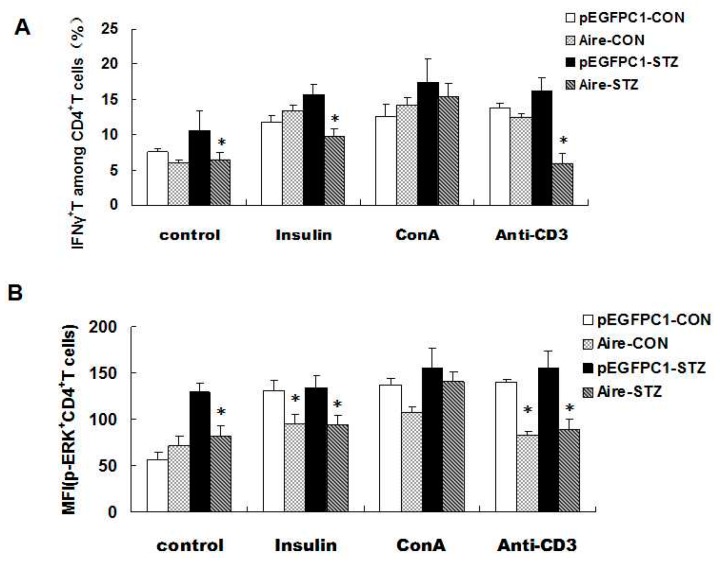
Adoptive transfer of Aire cells-co-cultured splenocytes suppressed autoreactive T cell activation by interfering with TCR signaling. Splenocytes from WT mice were co-cultured with Aire or control cells for 48 h. Then, the lymphocytes were isolated and injected into WT mice by tail vein followed by the induction of diabetes with STZ. The splenocytes were harvested and stimulated with insulin, ConA or CD3 antibody for 48 h prior to detection of the IFN-γ expression levels (**A**); and p-ERK expression levels (**B**) in CD4^+^ T cells by FACS. The results are presented as the mean values ± SD. Each experiment was repeated three to five times, and each group was compared with the pEGFPC1 group; * *p* < 0.05, Aire *vs.* pEGFPC1.

Finally, we investigated the expression of IFN-γ and p-EPK in CD4^+^ T cells simulated with an anti-CD3 antibody, ConA and insulin. The results demonstrated that the expression of IFN-γ and p-EPK after anti-CD3 antibody and insulin treatment was significantly lower in CD4^+^ T cells from mice transplanted with splenocytes from the Aire group. Moreover, no significant difference was detected between the Aire group and the control group following ConA stimulation (Figure 5A,B), indicating that the two groups of splenocytes did not differ in cell viability. Taken together, our data suggested that Aire cell-induced splenocytes delayed the occurrence of T1D in STZ-induced mice.

### 2.5. Discussion

*Aire* is primarily expressed in mTECs in the central immune system. Aire induces central tolerance by regulating the expression of the TRA genes and inducing the negative selection of self-reactive T cells and the production of regulatory T cells [2,10,11,12]. In the periphery, *Aire* is mainly expressed on DCs. However, the function and significance of Aire in the periphery remain unclear [9,17,18,19]. The present study primarily explored the effects of *Aire* expression in peripheral DCs on the function and activity of peripheral CD4^+^ T cells.

DCs can be divided into two major types based on their maturation status: immature and mature DCs. Immature and mature DCs display different phenotypes. Moreover, DCs with different maturation states exert distinct effects on T cells. Mature DCs induce effective immune responses by expressing costimulatory molecules and by highly expressing MHC II. Immature DCs often lack costimulatory molecules, express low levels of MHC II and exert immunosuppressive activities. Studies have shown that extrathymic Aire-expressing cells (eTACs) expressing low levels of CD80 and CD86 may serve as tolerant cells to induce antigen-specific immune tolerance. Additionally, stimulation of Toll-like receptors (TLRs) failed to reverse eTAC-induced immune tolerance [34,35,36]. The above findings are consistent with our results obtained using Aire-overexpressing DC2.4 cells. The present study showed that both Aire-overexpressing DC2.4 cells and control cells expressed low levels of costimulatory molecules (CD40, CD80, CD83, CD86, and CD11c) and MHC II-like molecules, which was consistent with the phenotypic characteristics of immature DCs. DC maturation following treatment with the DC activator LPS was accompanied by the upregulation of the expression of costimulatory molecules and MHC II-like molecules on the cell surface, which was consistent with previous reports [37]. However, the expression levels of costimulatory molecules and MHC II on the surface of the Aire cells were downregulated compared to the control cells after administration of the DC activator. Therefore, we speculated that Aire inhibited the expression of costimulatory molecules and MHC II in activated DCs, thereby maintaining the immature state of DCs and playing a role in maintaining peripheral immune tolerance.

One study showed that DCs present in the periphery also expressed TRAs and played a role similar to mTECs [38] in which they induced the clearance of autoreactive T cells and prevented the incidence of autoimmune diseases. Aire^+^ DCs expressed a high level of Ins2 compared to the total mouse splenocytes [22]. Therefore, we speculated that the expression of Aire in peripheral DCs affected peripheral immune tolerance by regulating the expression of TRAs. Our results showed that Aire-transfected DCs significantly enhanced the expression of T1D-associated TRAs, such as GAD65/67, Ins2, IA-2 and IGRP. These data demonstrated that Aire expression in peripheral DCs might play a role similar to its role in the central immune system. Thus, Aire induced the clearance of peripheral autoreactive T cells and participated in immune tolerance through the regulation of the expression of TRAs on DCs.

Previous reports have shown that the *production of* significant levels of IFN-γ by T cells is the main cause of autoimmune diseases such as T1D. The peripheral immune system maintains a state of tolerance by restricting IFN-γ^+^ T cells to a low percentage of total CD4^+^ T cells [39,40,41]. In the present study, splenocytes derived from STZ-T1D mice and WT mice were co-cultured with Aire-overexpressing DCs and then stimulated with insulin, which resulted in a significant reduction in the number of CD4^+^ IFN-γ^+^ T cells among the total splenocytes. These results indicated that Aire-overexpressing DCs played a role in preventing the occurrence of T1D by inhibiting the production of IFN-γ by CD4^+^ T cells or inducing the apoptosis of IFN-γ-producing autoreactive CD4^+^ T cells.

CD4^+^ T cells recognize specific antigens presented by MHC II molecules through TCRs and then receive stimulatory signals from the engaged TCRs. In the presence of synergistic actions of costimulatory molecules, TCR signaling pathways will be fully activated and further promote the production and release of downstream cytokines, such as IFN-γ, thereby eliciting immune responses. Loss of costimulatory molecules leads to the inactivation of TCR signaling pathways [42]. The results of our previous experiments showed that Aire inhibited the expression of costimulatory molecules and MHC II during the process of DC activation. Therefore, we investigated whether the Aire-overexpressing DC-induced reduction in the number of CD4^+^ IFN-γ^+^ T cells in mouse splenocytes was caused by the inactivation of TCR signaling pathways in CD4^+^ T cells. In the present study, splenocytes derived from STZ-T1D and WT mice were co-cultured with Aire-overexpressing DCs and then stimulated with insulin. The results showed that the levels of key molecules of the TCR signaling pathways (Ca^2+^ and p-ERK) were significantly decreased in STZ-T1D and WT mouse-derived CD4^+^ T cells after induction with Aire-overexpressing DCs. These results suggest a mechanism in which Aire suppresses the activity of CD4^+^ T cells by inhibiting the expression of costimulatory molecules during the process of DC activation and inhibiting TCR signaling pathways in CD4^+^ T cells, eventually leading to a reduction in the number of CD4^+^ IFN-γ^+^ T cells.

The expression of TRA genes in the thymus provides an opportunity for developing T cells to contact peripheral self-antigens and induces the apoptosis and clearance of CD4^+^ T cells that exhibit high affinity for the TRAs [43,44,45]. The above phenomenon also exists in the periphery [3,4]. The results of our previous experiments showed that DCs transfected with Aire exhibited significantly upregulated expression of T1D-related TRAs. Therefore, we examined CD4^+^ T cell apoptosis in STZ-T1D and WT mouse-derived splenocytes after co-culturing with Aire-overexpressing DCs. Our results were consistent with previous reports in the literature that demonstrated that Aire-transfected DCs significantly enhanced the apoptosis of STZ-T1D mouse-derived CD4^+^ T cells, whereas they exerted no significant effect on WT mouse-derived CD4^+^ T cells. These results indicated that Aire induced the apoptosis of autoreactive CD4^+^ T cells by promoting the expression of TRAs in DCs, which may represent another mechanism for the maintenance of immune tolerance. However, one cannot rule out the possibility that Aire-expressing cells may induce CD4^+^ T cell tolerance through other means, such as direct cell-cell contact or soluble mediators. We have measured the level of some soluble mediators, such as TGF-β was upregulated in Aire overexpressing cells. This may contribute to this effect. We also tried to investigate the effect using transwell cocultures, but unexpectedly there was no change in the number of regulatory T cells. We therefore think this effect may also depend on direct cell-cell contact. We will further investigate the other mechanisms involved the effect of Aire overexpression in our future study. In summary, Aire-overexpressing DCs maintain CD4^+^ T cell tolerance by inducing the functional inactivation and apoptosis of autoreactive CD4^+^ T cells.

Other studies have reported that T1D is caused by autoreactive T cells. Once activated, autoreactive T cells enter into pancreatic tissues, form inflammatory infiltrates and produce large amounts of IFN-γ, which leads to the further destruction of pancreatic β cells. Eventually, pancreatic β cells lose the ability to secrete insulin [40,41]. In a previous study, Aire was transfected into bone marrow cells. Aire was shown to promote the expression of TRAs in bone marrow cells, such as myelin oligodendrocyte glycoprotein (Mog). Moreover, adoptive transfer of Aire-overexpressing bone marrow cells delays the occurrence of experimental autoimmune encephalomyelitis (EAE) in Mog-induced mice [21]. Previously, we demonstrated that DCs transfected with Aire induced the inactivation of CD4^+^ T cells using *in vitro* assays. To investigate whether CD4^+^ T cells were capable of resisting activation after the induction of tolerance and truly played a role in preventing the occurrence of T1D, splenocytes derived from WT mice were *co-cultured* with Aire-overexpressing DCs and then adoptively transferred into WT mice. Then, the mice were induced with STZ. The blood glucose levels were significantly reduced in mice adoptively transferred with Aire-overexpressing DC-induced splenocytes. Furthermore, H&E staining of mouse pancreatic tissues showed a low degree of inflammatory infiltration. The IFN-γ and p-EPK expression levels were markedly reduced in CD4^+^ T cells among the total splenocytes following insulin stimulation. These results indicated that CD4^+^ T cells induced with Aire-overexpressing DCs were capable of delaying the occurrence of T1D in STZ-induced mice.

## 3. Experimental Section

### 3.1. Cells and Animals

The dedritic cell line DC2.4 was established by transducing granulocyte-macrophage CSF into *C57BL/J6* mice bone marrow cultures followed by supertransfection with myc and raf oncogenes [46], obtained from the Shanghai Cell Research Institute and cultured in 10% NCS-RPMI-1640 (Hyclone, Logan, UT, USA, Gibco-BRL). The DC2.4 cells were transfected with the pEGFPC1/Aire (Aire cells) or pEGFPC1 plasmids (pEGFPC1 or control cells in the following text), and stable cell lines were obtained following G418 selection as previously described [47]. C57BL/J6 mice were purchased from the Experimental Animal Center of Jilin University, and all mice were housed under specific pathogen-free conditions. The experimental procedures were approved by the Ethics committee of College of Basic Medical Sciences, Jilin University (Approval No.: 2014-96, Approval date: January, 2014).

### 3.2. Co-Culture of the DC2.4 Cell Line with Splenocytes

The spleens were removed from the *C57BL/J6* mice and gently dissociated into single-cell suspensions. Aire cells or pEGFPC1 cells (2 × 10^5^ per well) and splenocytes (4 × 10^6^ per well) were seeded into six-well plates. Once the cells attached to the dishes, 5 mg/mL ConA (Sigma-Aldrich, St. Louis, MO, USA), 10 mg/mL insulin (Dong Bao Pharm, Tonghua, Jilin, China) or 5 μg/mL anti-CD3 antibody (Tianjin Sungene Biotech, Tianjin, China) were added and the plates were incubated for 48 h. Finally, the splenocytes were harvested for flow cytometry and real-time PCR analyses.

### 3.3. RNA Isolation and Quantitative Real-Time PCR

Total RNA was extracted from the harvested splenocytes as described above or from the Aire and pEGFPC1 cells using RNAiso™ PLUS (Takara, Tokyo, Japan) and dissolved in DEPC-treated water. The amount of total RNA was measured using an ultraviolet spectrophotometer (Biotek, Burlington, VT, USA). cDNA was synthesized from 1.0 μg of total RNA using the M-MLV reverse transcriptase and oligo(dT) in a total volume of 20 μL according to the manufacturer’s instructions (Takara). Real-time quantitative PCR (qPCR) was performed using an ABI PRISM 7300 sequence detection system (Applied Biosystems, Carlsbad, CA, USA) with SYBR Premix Ex Taq™ II (Takara) following the manufacturer’s suggested protocol and using the following conditions: 95 °C for 30 s and 40 cycles of 95 °C for 5 s and 60 °C for 30 s. The results were analyzed using the formula 2^−∆∆Ct^. The following primers were used: GAPDH (forward: 5′-GACTTCAACAGCAACTCCCACTC-3′; reverse: 5′-TAGCCGTATTCATTGTCATACCAG-3′), Gad67 (forward, 5′-CTCAGGCTGTATGTCAGATGTTC-3′; reverse, 5′-AAGCGAGTCACAGAGATTGGTC-3′), IGF-2 (forward, 5′-GGCCCCGGAGAGACTCTGTGC-3′; reverse: 5′-GCCCACGGGGTATCTGGGGAA-3′), GAD65 (forward: 5′-TCAACTAAGTCCCACCCTAAG-3′; reverse: 5′-CCCTGTAGAGTCAATACCTGC-3′), IA-2 (forward: 5′-GATTCCCTTGGGTTTGTAGTTC-3′; reverse: 5′-TCCCT CCCTTCAGGTTTGA-3′), Ins2 (forward: 5′-ACCTTCAGACCTTGGCACTG-3′; reverse: 5′-GCTGGGTAGTGGTGGGTCTA-3′), chrA (forward: 5′-ACCTTCAGACCTTGGCACTG-3′; reverse: 5′-AAGCCTCTGTCTTTCCATC-3′), IGRP (forward: 5′-GTTCGGTATTGACCTGCTGTG-3′; reverse: 5′-TTGATGAAGCGATAAAGTTGC-3′, CD73 (forward: 5′-CAAATCCCACACAACCACTG-3′; reverse: 5′-TGCTCACTTGGTCACAGGAC-3′), Lag3 (forward: 5′-AGTGACTCCCAAATCCTTCG-3′; reverse: 5′-CCTCCTGAATCTCCAGCACA-3′), FR4 (forward: 5′-TGTCGCAAGCACTTCATCCA-3′; reverse: 5′-CGCCACCAGTCCTCACAATC-3′), PD-1 (forward: 5′-AGTGGGTATCCCTGTATTGC-3′; reverse: 5′-CTCCTCCTTCAGAGTGTCGT-3′), and IL-2 (forward: 5′-CATTGACACTTGTGCTCCTTGT-3′; reverse, 5′-TCCTGTAATTCTCCATCCTGCT-3′).

### 3.4. Flow Cytometry Analysis

The cells were collected and counted, and 1 × 10^6^ cells were suspended in 100 µL of PBS. The cells were incubated with PE-anti-CD40, PE-anti-CD80, PE-anti-CD83, PE-anti-CD86, PE-anti-Cd11C, PE-anti-MHC II and PE-Cy7-anti-CD4 (eBioscience, San Diego, CA, USA) on ice for 45 min and subsequently fixed with fixation/permeabilization concentrate with diluent (eBioscience) for 1 h. Alternatively, the cells were incubated with Fluo-3 for 30 min at room temperature for Ca^2+^ staining. Then, the cells were treated with 0.1% saponin (Sigma-Aldrich, St. Louis, MO, USA) and anti-IFN-γ-PE, anti-phosphorylated-ERK, and anti-IgG-PE (eBioscience, San Diego, CA, USA) at 4°C for 1 h. Next, the cells were washed with PBS and resuspended in 2% paraformaldehyde for analysis using a BD FACS Calibur flow cytometer.

### 3.5. Streptozotocin (STZ)-Induced Type 1 Diabetic Mouse Model

C57BL/6J mice were treated by intraperitoneal (i.p.) injection of STZ (80 mg/kg) dissolved in cold 0.1 M sodium citrate buffer solution once a day for five consecutive days [48]. The onset of diabetes was evaluated by measuring blood glucose levels in serum collected from the tail vein using an AccuChek system (Beyotime Biotechnology, Shanghai, China).

### 3.6. Adoptive Transfer of Splenocytes into Mice

After co-culture with the DC2.4 cell line as described above, 2 × 10^6^ splenocytes were intravenously (i.v.) injected into the tail veins of 8-week-old mice, once every 3 days and repeated for three times. Two days after the first transfer of splenocytes, the mice were induced by STZ as described above and observed for the onset of diabetes by measuring blood glucose levels.

### 3.7. Immunohistochemical Analysis

The pancreas was removed from mice and fixed in formalin, embedded in paraffin, sectioned, and stained with H & E.

### 3.8. Statistical Analysis

All experimental data are reported as the means, and the error bars represent the experimental standard errors. Statistical analysis was performed using Student’s *t*-test. The statistical significance between two groups was set at *p* < 0.05.

## 4. Conclusions

In conclusion, our study shows that Aire-overexpressing DCs play a role in inducing the functional inactivation and apoptosis of autoreactive CD4^+^ T cells. Our data suggest that peripheral CD4^+^ T cell tolerance can be maintained through the combined effects of the two mechanisms and indicate that approaches to modulate Aire expression in DCs could be a potential method to control autoimmunity.

## Supplementary Materials

Supplementary materials can be found at http://www.mdpi.com/1422-0067/17/1/38/s1.
