# Supplementary material for: Aire-Overexpressing Dendritic Cells Induce Peripheral CD4+ T Cell Tolerance
Source: Int J Mol Sci. 2015 Dec 29;17(1):38. doi: 10.3390/ijms17010038 (PMC4730283; doi:10.3390/ijms17010038)
Supplement: Supplementary file 1 [file ijms-17-00038-s001.pdf]

# Supplementary Materials: Aire-Overexpressing Dendritic Cells Induce Peripheral CD4⁺ T Cell Tolerance

**Dongbei Li, Haijun Li, Haiying Fu, Kunwei Niu, Yantong Guo, Chuan Guo, Jitong Sun, Yi Li and Wei Yang**

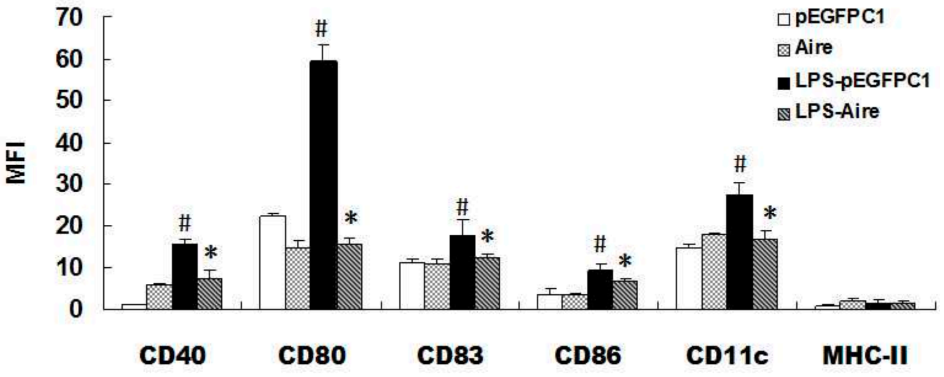


**Figure S1.** The expression of CD40, CD80, CD83, CD86, CD11c and MHC II were detected by FACS in Aire and control cells with or without stimulation with 10 μg/mL of LPS for 24 h; # $p < 0.05$, LPS stimulation *vs.* control; * $p < 0.05$, Aire *vs.* pEGFPC1.

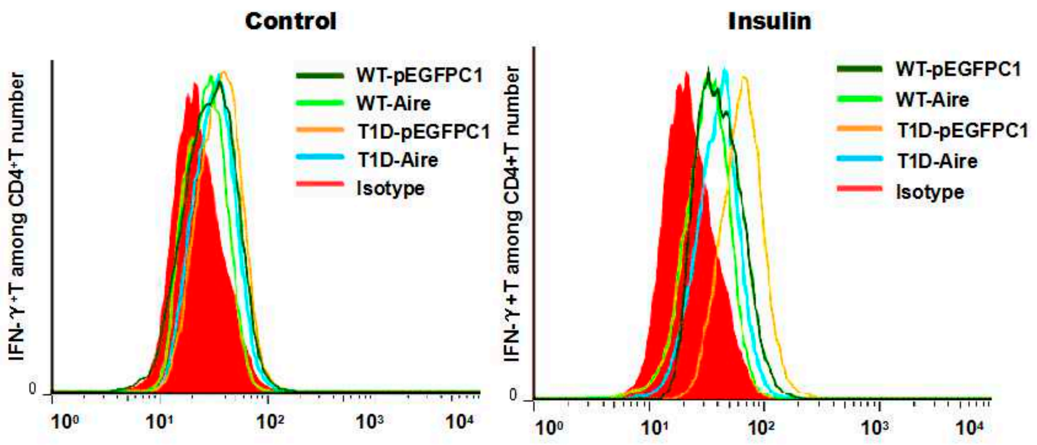


**Figure S2.** Splenocytes from STZ-induced T1D mice or WT mice co-cultured with Aire or control cells for 48 h in the presence of insulin. The IFN-γ expression levels in CD4⁺ T cells were examined by FACS.

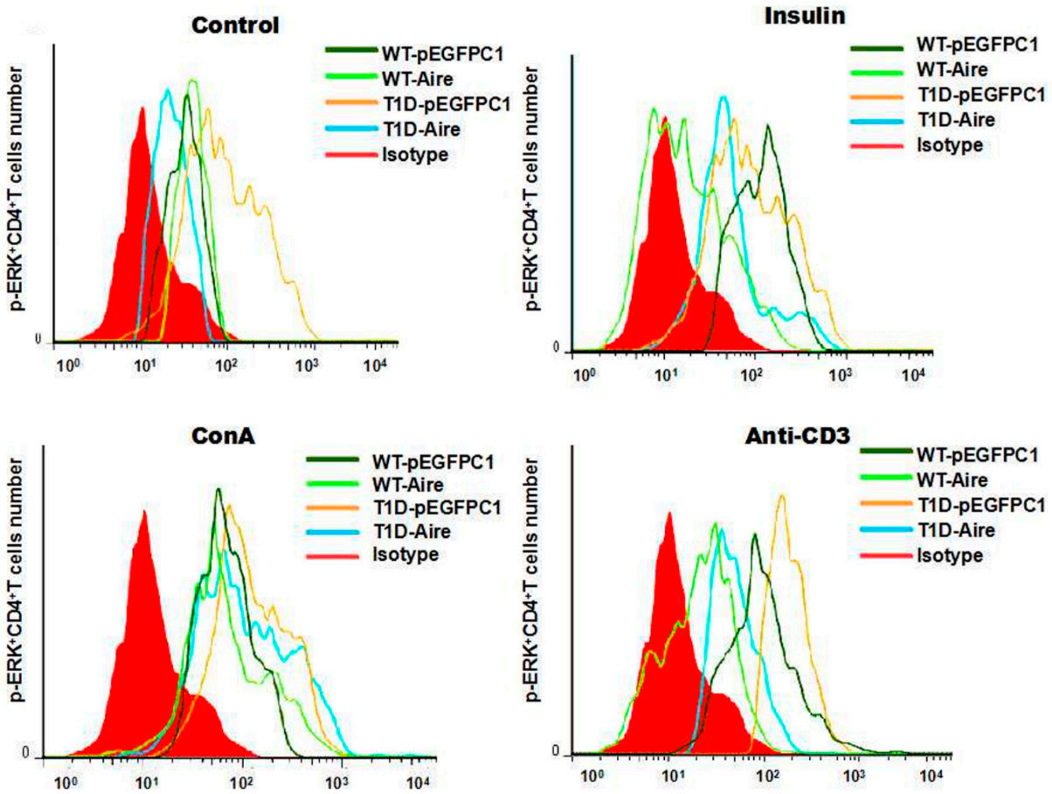


(**A**)

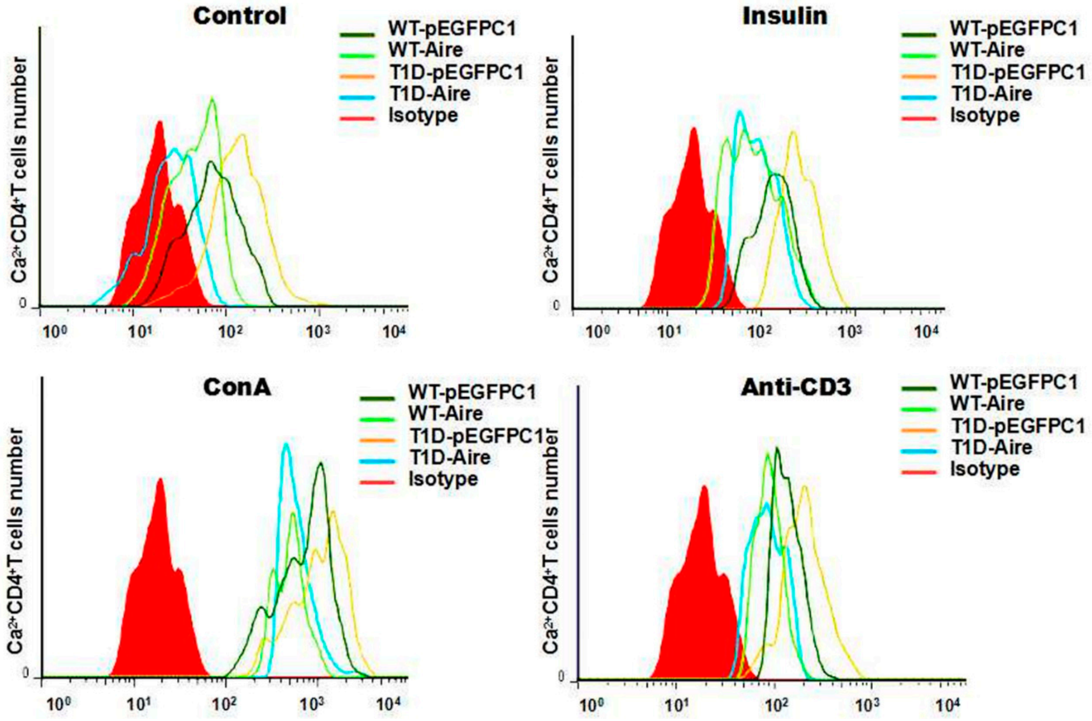


(**B**)

**Figure S3.** (**A**) The p-ERK expression levels; and (**B**) the concentration of $Ca^{2+}$ in $CD4^{+}T$ cells were examined by FACS.

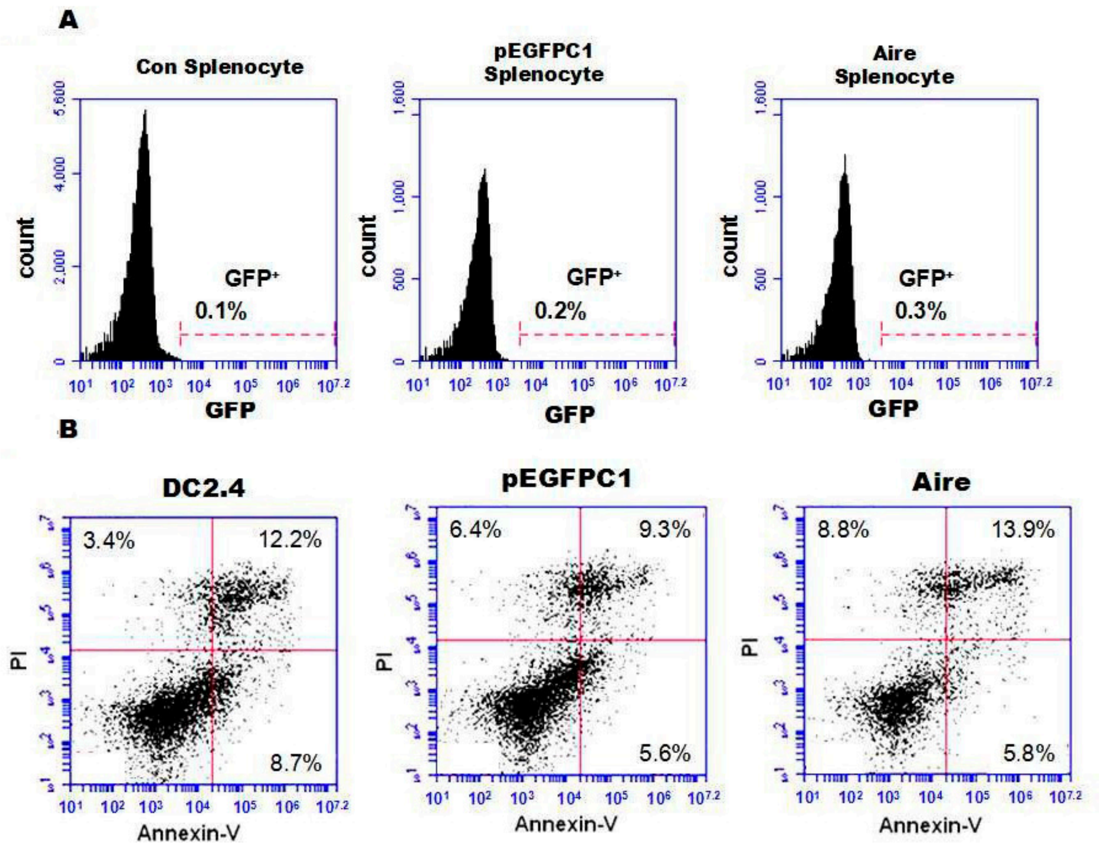


**Figure S4.** (**A**) Percentage of GFP+ cells in splenocyte isolated from the co-cultured medium. (**B**) The % of apoptotic T-cells which co-cultured with DC cells.
